# SymbiQuant: A Machine Learning Object Detection Tool for Polyploid Independent Estimates of Endosymbiont Population Size

**DOI:** 10.3389/fmicb.2022.816608

**Published:** 2022-05-19

**Authors:** Edward B. James, Xu Pan, Odelia Schwartz, Alex C. C. Wilson

**Affiliations:** ^1^Department of Biology, University of Miami, Coral Gables, FL, United States; ^2^Computational Neuroscience Lab, Department of Computer Science, University of Miami, Coral Gables, FL, United States

**Keywords:** endosymbiont quantification, symbiosis, aphid, *Buchnera*, neural network, computer vision

## Abstract

Quantifying the size of endosymbiont populations is challenging because endosymbionts are typically difficult or impossible to culture and commonly polyploid. Current approaches to estimating endosymbiont population sizes include quantitative PCR (qPCR) targeting endosymbiont genomic DNA and flow-cytometry. While qPCR captures genome copy number data, it does not capture the number of bacterial cells in polyploid endosymbiont populations. In contrast, flow cytometry can capture accurate estimates of whole host-level endosymbiont population size, but it is not readily able to capture data at the level of endosymbiotic host cells. To complement these existing approaches for estimating endosymbiont population size, we designed and implemented an object detection/segmentation tool for counting the number of endosymbiont cells in micrographs of host tissues. The tool, called SymbiQuant, which makes use of recent advances in deep neural networks includes a graphic user interface that allows for human curation of tool output. We trained SymbiQuant for use in the model aphid/*Buchnera* endosymbiosis and studied *Buchnera* population dynamics and phenotype over aphid postembryonic development. We show that SymbiQuant returns accurate counts of endosymbionts, and readily captures *Buchnera* phenotype. By replacing our training data with data composed of annotated microscopy images from other models of endosymbiosis, SymbiQuant has the potential for broad application. Our tool, which is available on GitHub, adds to the repertoire of methods researchers can use to study endosymbiosis at the organismal, genome, and now endosymbiotic host tissue or cell levels.

## Introduction

To understand the evolution and ecology of eukaryotic hosts and their microbial partners it is necessary to study the population dynamics of both host and symbiont. The eukaryotic cell itself is a product of a symbiosis between an α-proteobacteria and an archaea (Cox et al., 2008; Koonin, 2015; Zaremba-Niedzwiedzka et al., 2017). Notably, symbiotic interactions between eukaryotes and microbial partners, both beneficial and deleterious, continue to impact eukaryotic, and microbial evolution (Engelstädter and Telschow, 2009; Rockwell et al., 2014; Radzvilavicius and Blackstone, 2015; Moelling and Broecker, 2019). Some symbiotic partnerships are pairwise like that of the Hawaiian bobtail squid, *Euprymna scolopes*, with the bioluminescent bacteria, *Vibro fisheri* (Nyholm and McFall-Ngai, 2021). In contrast, other symbiotic partnerships are more dynamic involving a single eukaryotic host interacting with a complex microbial community such as that found in the rumen of a cow (Weimer, 2015).

The size of symbiont populations can dramatically impact the fitness of symbiotic partners (Bronstein, 1994). Some mutualisms feature complex molecular mechanisms that regulate population size; for example in the squid/*Vibrio* symbiosis, *Vibrio* have evolved multiple quorum-sensing systems that regulate the expression and translation of bioluminescence genes (Verma and Miyashiro, 2013). Or in symbioses that are parasitic or pathogenic it is typical to observe density dependent relationships between host and symbiont (Knell et al., 1998; Råberg et al., 2007). For example, the gram-positive bacterium *Staphylococcus aureus* has two modes of infection in humans; sessile, where *S. aureus* produce adhesins that maintain localized infections, and flotile, where *S. aureus* stop producing adhesins, and start producing lytic enzymes and virulence factors that promote systemic infection (Yarwood and Schlievert, 2003). The change in infection mode from sessile to flotile is governed by quorum sensing mechanisms such that *S. aureus* population density dictates the strength of the pathogenic relationship between *S. aureus* and humans (Novick, 2003). Given that symbiont population size can dramatically affect the nature of a symbiosis it is necessary to study the population dynamics of hosts and their symbionts to understand the ecology and evolution of symbiosis. Unfortunately counting microbial symbionts is often challenging because the symbionts are both microscopic and unculturable.

To date several approaches to counting and estimating the population size of microbial symbionts have been developed. The most common method has involved application of quantitative-PCR (qPCR) (Simoncini et al., 2001; Kaech and Vorburger, 2020; Bodenhausen et al., 2021). A limitation of qPCR quantification is that it measures the number of endosymbiont genome copies, rather than the number of endosymbiont cells (Neiers et al., 2021). Microbial endosymbionts are frequently highly polyploid and levels of polyploidy are known to vary across different conditions (Komaki and Ishikawa, 2000; Mergaert et al., 2006; Woyke et al., 2010). Therefore, population estimates based on genome copy number can be confounded by mismatches between the number of genomes and the number of bacterial cells in endosymbiont populations. That said, genome copy numbers can be useful because they provide information for understanding the evolution and function of endosymbionts (Viñuelas et al., 2011; Van Leuven et al., 2014; Engl et al., 2018). A second common approach to counting symbionts and estimating symbiont population size has involved use of microscopy coupled with manual counts of symbionts (e.g., Mira and Moran, 2002) or even application of basic image analysis tools such as thresholding and particle counting (Serbus et al., 2015). While microscopy based approaches are not confounded by variable ploidy, analysis of large datasets is infeasible with respect to manual counting, and automated approaches like thresholding and particle counting only work in systems where symbionts are not tightly clustered and overlapping (see e.g., Serbus et al., 2015). Disappointingly, thresholding, and particle counting approaches do not work when applied to the tissues of symbiotic organs in which microbial populations are tightly packed (Douglas, 1989). A third approach has used flow cytometry to count endosymbionts (Simonet et al., 2016; Takahashi, 2016). Flow cytometry techniques directly count endosymbiont cells and allow for the generation of large insect-level datasets (Simonet et al., 2016). However, current approaches involve dissociating endosymbionts from their endosymbiotic tissue, which means for some systems it is not possible to capture important data at the level of the host cell. Given the strengths and shortcomings of the available approaches for estimating microbial symbiont population size we set out to add a new approach to the toolkit for quantification of endosymbiont populations.

**FIGURE 1:**
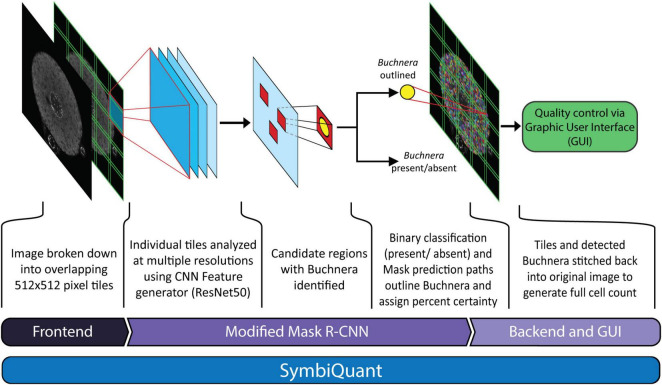
Network architecture of SymbiQuant. At the frontend input images are sectioned into overlapping tiles. The *Buchnera* detection/segmentation network which is based on Mask R-CNN is run on separate tiles, which are then recombined into final outputs. A Graphic User Interface allows user validation of type I and II errors.

Here we present and test SymbiQuant, a high throughput tool for estimating microbial population size based on endosymbiont cell counts from microscopy images (Figure 1). To realize our approach our interdisciplinary team of biologists and computer scientists leveraged a neural network object-detection tool to process microscopy images of host symbiotic tissues. Briefly, we generated microscopy images of host symbiotic tissues that we passed to a modified Mask Region-based Convolutional Neural Network (Mask R-CNN) trained to identify and count our focal endosymbiont. In order to improve object prediction, it was necessary to build front and backends to the pipeline that allowed at the front end for the reduction of image complexity, and that allowed at the back end for human curation to facilitate resolution of type I and II errors. SymbiQuant can be trained to count endosymbionts in diverse symbiotic systems through development of system-specific training sets. Here, we demonstrate application of SymbiQuant in the model aphid/*Buchnera* endosymbiosis through analysis of *Buchnera* population dynamics across aphid post-embryonic development. The aphid/*Buchnera* symbiosis, like that of many intracellular symbioses is ancient (McCutcheon et al., 2019). *Buchnera* have been vertically transmitted from mother to progeny for 150 million years (Dohlen and Moran, 2000). For most of an aphid’s life *Buchnera* are housed inside specialized aphid cells called bacteriocytes, that aggregate with another cell type called sheath cells to form a bilobed organ, the bacteriome. Using SymbiQuant we studied *Buchnera* populations in intact bacteriocyte cells from the aphid *Acyrthosiphon pisum* at the second, third and fourth larval instar, and four time points during adulthood.

## Methods

### Aphid Rearing and Sampling

*Acyrthosiphon pisum* line LSR1 was raised on *Vicia faba* at 20°C under a 16-h light/8-h dark cycle. Following the time points of Simonet et al. (2016) we generated age structured cohorts of aphids by placing 3–5 asexual adult females on a single potted *V. fabae* for 24-h before removing the adults and collecting progeny at day 3 (2nd instar), day 5 (3rd instar), day 7 (4th instar), and days 9, 13, 16, and 23 (adult). By day 23 the reproductive output and health of LSR1 aphids were in decline.

### Bacteriocyte Preparation and Imaging

Bacteriocytes from multiple asexual females were dissected in a single well of a 9-well Pyrex spot plate into ice cold 0.2% triton in 1× PBS (PTx) using dissection forceps and a 00-gauge minuten pin embedded and glued into the end of a wooden chopstick. Briefly, we gripped aphids around their prothorax using the dissection forceps to submerge them and then cut open the abdomen using the minuten pin tool. We released all the internal tissues and embryos by gently shaking the body of the aphid before removing the body from the dissection dish. In order to image sufficient numbers of isolated, intact bacteriocytes it was necessary to dissect multiple individuals into a single spot plate well. For 2nd instar aphids, we dissected 20–50 individuals, for 3rd and 4th instars, we dissected 20 individuals, and for adults we dissected 15 individuals.

Next we performed the dissociation, fixing and staining of dissected tissues at room temperature in the dissection well covered by an opaque box, on an orbital shaker at 60 rpm. All washes and buffer changes were done slowly under a dissecting microscope using a pasteur pipette which had been rinsed in PTx prior to use to remove any glass dust. In order to perform the washes very carefully it was necessary to seal the rubber bulb to the glass pipette using parafilm. To maximize the number of bacteriocyte cells recovered, all steps were carried out in the same spot plate well. To dissociate bacteriocyte cells and facilitate the imaging of individual bacteriocytes we first performed a 20 min ice cold 0.025% trypsin incubation, followed by two 15 min washes in ice cold PTx, and one 15 min wash in ice cold 1.8% paraformaldehyde in PTx. To fix tissues we incubated tissues in ice cold 3.6% paraformaldehyde in PTx for 25 min, followed by three 15 min washes in ice cold PTx. To stain DNA and actin we incubated tissues in ice cold 1 μg/ml DAPI and 0.5 μg/ml phalloidin-TRITC in PTx for 90 min, followed by three 15 min washes in ice cold PTx.

Following staining we carefully sorted bacteriocytes from embryos, guts and other tissues. First we used forceps to remove embryos and guts. Next we used the forceps tip to create a gentle current in the wash buffer to sort bacteriocytes from fat body and other small cellular debris. Bacteriocytes were transferred in a line down the middle of a clean glass slide using a wide bore p200 pipette tip, set at 50 μl. We next distributed 25 μl of vectashield antifade mounting media (Vector Laboratories) down each side of the bacteriocytes before sealing them under a No. 1.5, 24 × 50 mm glass coverslip using nail varnish.

Cells were imaged using a Leica TCS SP5 confocal microscope in the University Miami, Department of Biology Confocal Microscopy Facility. Data for each cell was captured as a single slice through the cell (0.005μm thick) at the point at which the nucleus was visually evaluated to be the widest. The DAPI stain facilitated identification of the cell nucleus and *Buchnera*, while actin labeled by phalloidin-TRITC facilitated discrimination of heavily vacuolated bacteriocytes from ruptured cells. Ruptured bacteriocyte cells were excluded from our dataset. We imaged 74 bacteriocyte cells for the training and validation of the tool, and a further 126 to demonstrate SymbiQuant use.

### Preparation of Confocal Images for Model Training, Testing, and Validation

For each confocal image we exported the DAPI channel in .png file format with 2,048 × 2,048 or 1,024 × 1,024 pixels—note that lossy file formats should be avoided at this stage because they introduce unnecessary data compression and loss. Note also that images must be larger than 512 × 512 pixels for our tool to work. We outlined all intact *Buchnera* cells in the .png files using labelme (Wada, 2016). To complement our dataset, we performed the same annotation on cropped bacteriocyte images previously taken by Price et al. (2014) and Feng et al. (2019). Next we transformed the labelme output .json files into COCO format using labelme2coco (Zhang, 2019). To fit the images into RAM and GPU memory for training the network we cropped the 74 bacteriocyte cell images (.png files) and their corresponding annotation files (COCO format .json file) into non-overlapping 512 × 512 pixel tiles—every image tile had a paired annotation tile corresponding to the same image coordinates. In total we generated 674 non-overlapping image tiles that we randomly split into a training set of 614 tiles representing 40,153 *Buchnera*, and a validation set of 60 tiles representing 3,957 *Buchnera*. Additionally, to test the final model’s performance, we prepared a test set of 14 annotated full-cell images that were split into 350 tiles, 50 for each point of development representing a total of 13,725 *Buchnera*.

### Development of Mask Region-Based Convolutional Neural Network *Buchnera* Recognition Algorithm

Recent CNN-based algorithms, such as Mask Region-based Convolutional Neural Network (Mask R-CNN), have achieved success in real-life object detection and segmentation (He et al., 2020; Minaee et al., 2021). Given that *Buchnera* at the cellular level are dense, small and more regular than objects in natural images, we modified Mask R-CNN to better fit our needs. The modifications include development of a customized learning rate scheduler, customization of object anchor size and shape, modification of feature map resolutions, and data augmentation. We implemented the network using the Detectron2 library^1^ with a Pytorch backend^2^. Processing high resolution images such as those generated by confocal microscopy, requires high-end GPUs. To lower the GPU requirement, we developed a tiling pipeline, which crops images into smaller tiles for training and prediction and stitches tiles back together after the network has run through all the tiles. We used ResNet50 as a backbone, passed feature maps through the Feature Pyramid Network (FPN), and then on to the Region Proposal Network (RPN) (He et al., 2016, 2020; Lin et al., 2017). After passing regions of interest to a convolutional upsampling head, we removed regions of low likelihood using a set of detection criteria as described below.

**FIGURE 2:**
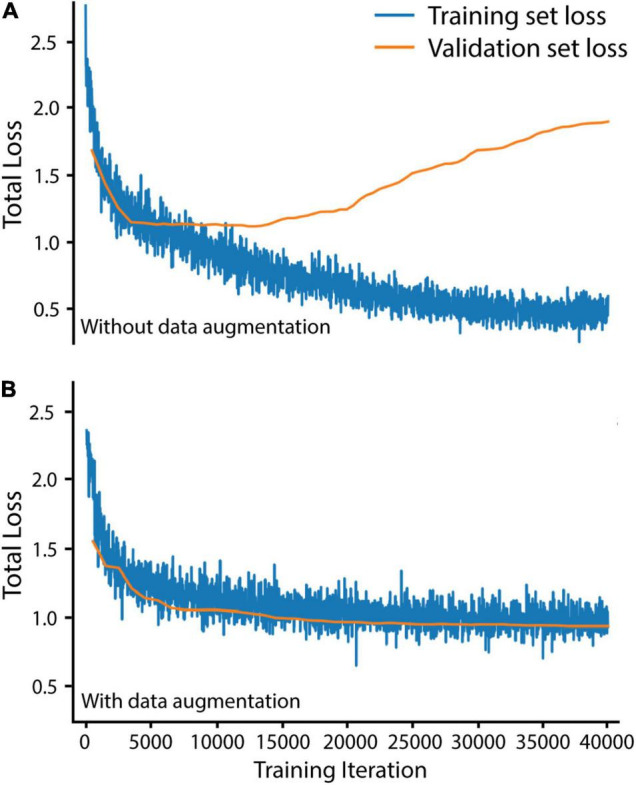
Data augmentation was necessary to avoid overfitting Mask R-CNN on our training set. **(A)** Training and validation loss without data augmentation. **(B)** Training and validation loss with data augmentation. Total loss is the sum of bounding box regression loss, ROI head classification loss, mask loss, region proposal network classification loss, and region proposal network localization loss. Training set loss was measured over 20 randomly selected training set tiles every 20 iterations. Validation set loss was measured over all 60 validation set tiles every 500 iterations.

We trained Mask R-CNN on our training and validation sets for 40,000 iterations, using the stochastic gradient descent optimizer with a warmup and cosine decay learning rate scheduler (implemented from Detectron2) to maximize improvements throughout training. To avoid overfitting the *Buchnera* prediction algorithm to our training set, we augmented our training set before training by randomly scaling brightness (0.6–1.8), contrast (0.6–1.8), scale (0.5–1), and performing random horizontal and vertical flips (code in https://github.com/WilsonLabMiami/SymbiQuant, “augmentation.py”). Data augmentation was necessary for achieving alignment of training and validation set loss — loss alignment indicates that the algorithm has not been overfitted (Figure 2). Next, we designed a *Buchnera* recognition network by adapting the default Mask R-CNN. Briefly, ResNet50 feature maps generated at each of the 2nd, 3rd, 4th, and 5th residual blocks were passed to the FPN, which combined them into four new hierarchical feature maps with 16 × 16, 32 × 32, 64 × 64, and 128 × 128 resolutions. Next, these new feature maps were passed to the RPN which identified anchors putatively containing *Buchnera* by consensus across multiple scales. Note that we did not use the feature map generated at the 6th residual block as occurs using the default settings of ResNet50 because *Buchnera* are small, dense, and regular in shape relative to other objects in the COCO dataset. In addition, because *Buchnera* have a toroidal shape in DAPI-stained confocal images, we constrained the FPN to search for regions with an aspect ratio of 1 (i.e., a square). Within the RPN, the anchor sizes were set to 32 × 32 pixels, 64 × 64 pixels, 128 × 128 pixels, and another at 128 × 128 pixels. Default anchors at 16 × 16 pixels and 256 × 256 pixels were not used because they were too small, or too large for *Buchnera* prediction. *Buchnera* predicted across all four feature map resolutions were marked as regions of interest with higher confidence than those predicted across only three resolutions, and so on. For each 512 × 512 tile up to 1,000 regions of interest could be identified to serve as anchors for the return of a *Buchnera* mask and bounding box; the number 1,000 was chosen because it is much larger than the number of *Buchnera* found in any 512 × 512 tile where the mean number of *Buchnera* identified by manual annotation was 66.7.

**FIGURE 3:**
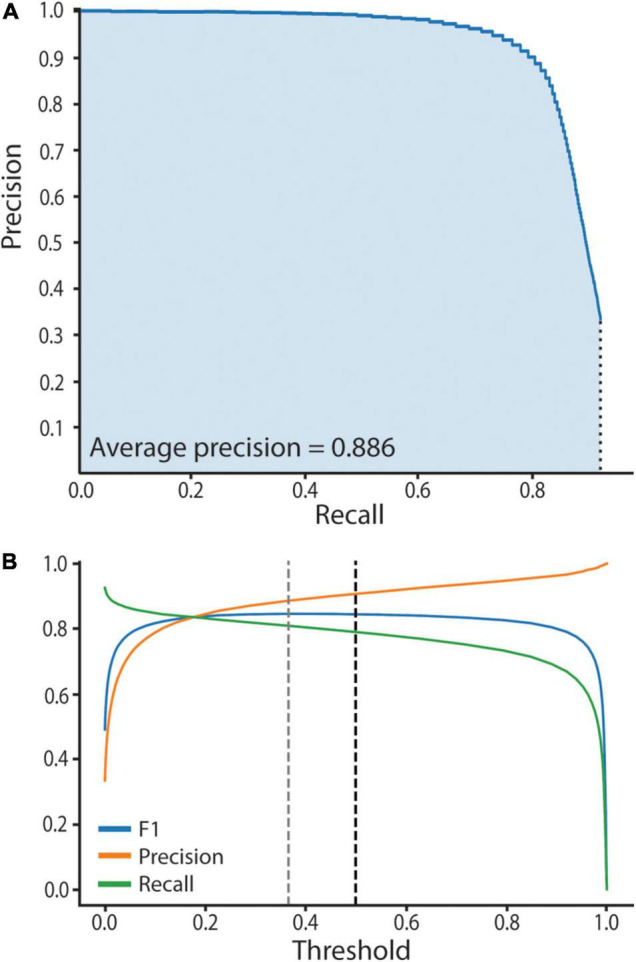
SymbiQuant accurately predicts *Buchnera* in confocal microscope images. **(A)** Precision-Recall Curve for SymbiQuant. The average precision score (AP) is the shaded area under the curve. We extrapolated to recall = 0, precision = 1. We did not extrapolate to recall = 1, precision = 0 as our model is unable to achieve recall = 1. **(B)** Precision, Recall, and F1 (the harmonic mean of precision and recall) for SymbiQuant’s *Buchnera* dataset, across all thresholds. Dashed black line shows the 0.5 threshold cutoff used in this study, dashed gray line shows the threshold that maximizes the F1 score—0.366).

We assessed neural network performance to empirically establish high confidence predictions. Computer vision neural network performance is typically assessed by computation of recall and precision metrics, where recall = the number of true positives divided by the number of manually annotated objects, and precision = the number of true positives divided by the total number of predictions. The harmonic mean of precision and recall gives the F1 score. A high F1 score reveals the network is making accurate predictions, while a low F1 score reveals inaccurate predictions. Specifically, to assess our trained network’s performance and empirically establish the model threshold we used a test set of 14 manually annotated bacteriocyte images, two from each age point. The test set of images was unseen and had not informed the training process. We computed three metrics: (1) A precision-recall curve, (2) an average precision score (AP), and (3) a F1 score. To do this we first had to compute prediction scores and assign predictions as true or false positives. Prediction scores ranged between 0.01 and 1, where a score of 0.01 equates to no similarity to *Buchnera* in the training set and a score of 1 equates to high similarity to *Buchnera* in the training set. Predictions were defined as “true positives” if they had an Intersection over Union (IoU) > 0.5 with a manually annotated *Buchnera*, and “false positives” if they did not have an IoU > 0.5 with any manually annotated *Buchnera*. To generate the precision-recall curve we used the test set that contains 14 images described in the previous subsection. We ordered the predictions scores from smallest to largest and separated them into 100 bins, each containing the same number of predictions. The maximum prediction score for each bin defines a threshold value. For each threshold value we calculated recall, precision, and F1 score. To calculate the AP score we plotted the precision-recall curve (AP = area under the precision-recall curve) (Figure 3).

Lastly, the RPN passed anchors with a prediction score higher than 0.5 to a 4-layer convolutional upsampling head (256 channels) that predicted a mask, and a 2-layer fully connected head (1,024 neurons) that returned bounding box coordinates.

### SymbiQuant

In order to utilize the MASK R-CNN *Buchnera* recognition algorithm on high resolution whole cell confocal images it was necessary to develop a frontend that split images into tiles, a backend that merged tiles back together, and a graphic user interface (GUI) that allowed for human curation (Figure 1). We call the assembled pipeline, *SymbiQuant*.

At the frontend, the input 2,048 × 2,048 pixel images were cropped to overlapping 5 × 5 tiles for analysis. The tiles were each 512 × 512 pixels, with an 85 pixel overlap between tiles. Each tile was then run through the MASK R-CNN *Buchnera* recognition algorithm.

At the backend, following analysis of the 25 tiles that comprised an image, we stitched the tiles back together. Predicted *Buchnera* that were smaller than 10% of the median size, and larger than five times the median size were removed as false positives. To avoid double counting within the 85-pixel-wide-areas-of-overlap we randomly dropped one of any two predicted *Buchnera* with an intersection over union (IoU) larger than 0.5 (code in project github).

To allow the removal of false positives and annotation of false negatives, we developed a GUI that overlays predicted *Buchnera* masks on the original input images (see manual and code in project github). The GUI outputs a “result” file that contains the coordinates of every *Buchnera* mask identified in its associated bacteriocyte image. See “Buchnera_metrics.py” in the project github for an example of how to use these result files in data analysis.

### Demonstration of SymbiQuant Use and Image Analysis

To demonstrate application and test performance of SymbiQuant across aphid post-embryonic development we imaged 16–22 individual bacteriocyte cells from each of seven time points that spanned the 2nd juvenile instar through adults in early senescence. To isolate sufficient intact bacteriocytes for statistical analysis we needed to perform between two and four imaging runs at each time point; typically more imaging runs were needed at the earlier time points. To analyze these images we ran SymbiQuant with GUI correction on each image, recording the number of *Buchnera* in a bacteriocyte and the area of each *Buchnera* cell (μm^2^). Additionally for each image we used the measure function in FIJI (Schindelin et al., 2012) to record the whole cell area (μm^2^) using the phalloidin-TRITC channel, and the nuclear area (μm^2^) using the DAPI channel. Lastly, we used “set scale” in FIJI to record the pixel:micron ratio of each image to convert from *Buchnera* pixel area to μm^2^ (Schindelin et al., 2012). Using these data for each image we calculated: (1) cytoplasm area (μm^2^) = whole cell area−nuclear area, (2) *Buchnera* density (*Buchnera.*μm^–2^) = number of *Buchnera* / cytoplasm area, (3) *Buchnera* cell area ((μm^2^) = *Buchnera* cell area in pixels^2^ * (pixel:micron ratio)^2^, (4) *Buchnera* diameter (μm) = √(*Buchnera* cell area / π), and (5) fraction of cell cytoplasm occupied by *Buchnera* (%) = (area of each *Buchnera* cell−area where *Buchnera* overlap with each other) / cytoplasm area. To analyze *Buchnera* cell areas we trimmed data with an absolute z-score greater than 3.

### Statistics

We analyzed all metrics for outliers, defining an outlier as a data point with an absolute z-score > 3. A single day 9 bacteriocyte was unusually large, and so we excluded this cell in the analysis of the following metrics: (i) *Buchnera* per bacteriocyte, (ii) percentage cytoplasm area occupied by *Buchnera*, (iii) *Buchnera* per μm^2^ cytoplasm, (iv) bacteriocyte nucleus area and (v) bacteriocyte area. Next, for each time point, we identified outliers in our *Buchnera* area dataset using the same z-score filter. We identified 10 *Buchnera* outliers at the second instar (0.08% of second instar dataset), 17 at the third instar (0.14% of third instar dataset), 344 at the fourth instar (1.7% of fourth instar dataset), 240 at day 9 adult (1.6% of day 9 dataset), 75 at day 13 adult (0.6% of day 13 dataset), 271 at day 16 adult (1.9% of day 16 dataset), and 552 at day 23 adult (4.9% of day 23 dataset). All outliers were removed from the datasets.

To compare metrics across aphid timepoints we checked for equal variance across samples using Levene’s test, and performed either an ANOVA with Tukey *post hoc* grouping for parametric data (percentage cytoplasm occupied by *Buchnera*), or a Kruskal-Wallis test with Dunn *post hoc* analysis for non-parametric data with Benjamini/Hochburg correction (all other metrics). We used Spearman correlations to investigate the relationship between aphid age and (i) bacteriocyte nucleus area, and (ii) *Buchnera* per μm^2^ of cytoplasm. To test for increasing variance in metrics with aphid age, we calculated the absolute difference from the mean for every datapoint in each of the four adult aphid timepoints (day 9–23). Differences in absolute values between timepoints were interrogated with Kruskal-Wallis tests with Dunn *post hoc* analysis for non-parametric data with Benjamini/Hochberg correction. To test for increase or decrease in metrics throughout adulthood, we performed linear regressions across the last four timepoints and recorded the slope with 95% confidence intervals [slope ± (1.96 × standard deviation)]. All statistical analyses were performed in python3 using “statsmodels_*posthoc*” and “scipy” packages.

## Results

### SymbiQuant Identifies *Buchnera* With High Accuracy

**FIGURE 4:**
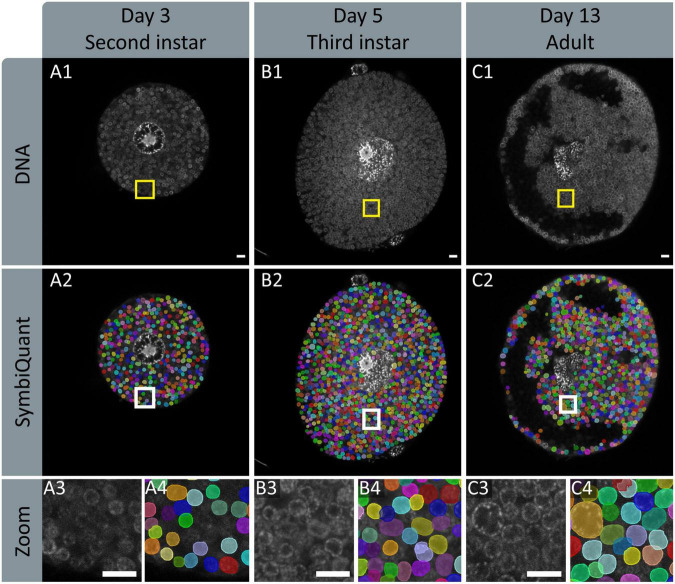
Input and output images of SymbiQuant. The top row shows DAPI channels of aphid bacteriocytes **(A1,B1,C1)**. The second row shows SymbiQuant output overlaid on the input image to show detected Buchnera **(A2,B2,C2)**. The third row shows the indicated zoomed section from the input **(A3,B3,C3)** and output **(A4,B4,C4)** images. Columns represent aphids at different age points, Day 3/second instar **(A)**, day 5/third instar **(B)**, and day 13/adult **(C)**. All Scale bars are 10 μm.

To facilitate high-throughput quantification of *Buchnera* populations in images of intact bacteriocyte cells we developed a machine vision tool (Figure 4). Our tool predicts *Buchnera* with both high precision and high accuracy (Figure 3A). For our recognition algorithm the probability that any one prediction from the network is a true positive as measured by precision was 0.885. While the fraction of real objects predicted by our model as measured by recall was 0.809. For our recognition algorithm, the average precision score (AP), a metric that captures precision and recall across all thresholds, was 0.886 (Figure 3A). While SymbiQuant is accurate, it does not achieve 100% recall or precision (Figure 3B). For this reason, in building the SymbiQuant pipeline, we included a GUI to enable human curation of model output.

### *Buchnera* Cell and Population Size Change Across Aphid Post-embryonic Development

**FIGURE 5:**
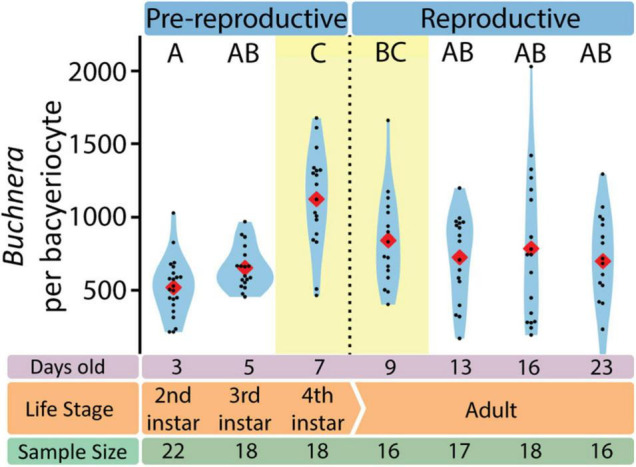
The number of *Buchnera* per bacteriocyte varies with aphid life stage. Letters indicate Dunn *post hoc* grouping with Benjamini/Hochberg correction. Red diamonds indicate means, black dots indicate individual data points.

**FIGURE 6:**
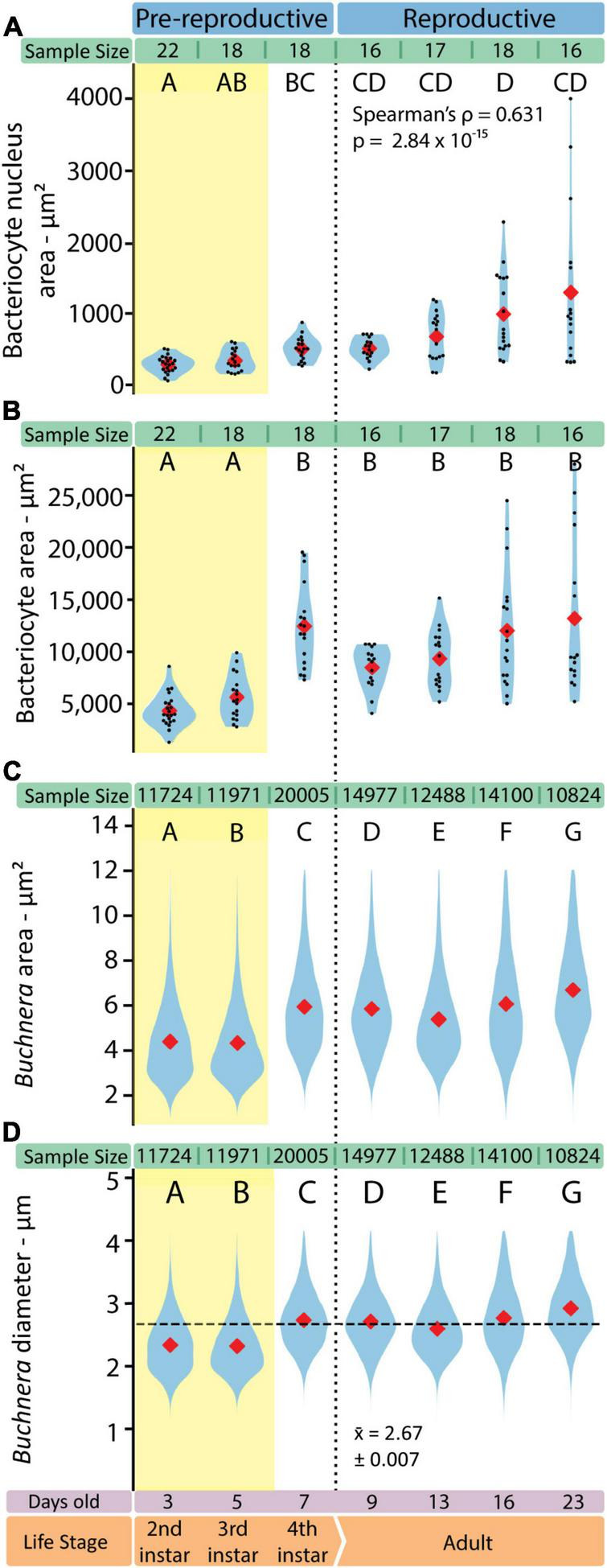
The shapes of both *Buchnera* and bacteriocytes change in consistent ways through aphid development. **(A)** Bacteriocyte nucleus area measured in FIJI. **(B)** Bacteriocyte cell area measured in FIJI. Sample sizes represent the number of full bacteriocyte images at each time point. **(C)** *Buchnera* cell areas measured by SymbiQuant. **(D)** *Buchnera* diameter, calculated from the area measured by SymbiQuant, with horizontal dashed line showing pooled mean diameter. In all violin plots red diamonds indicate means. When present, black dots indicate individual data points. Sample sizes are listed above each plot. Letters indicate Dunn *post hoc* grouping with Benjamini/Hochberg correction.

With SymbiQuant we measured the size of *Buchnera* populations inside single bacteriocytes. We found that *Buchnera* populations reach their maximum size immediately before host reproductive maturity (Figure 5). The number of *Buchnera* per bacteriocyte significantly increases between the third and fourth instar of development. At adulthood, the number of *Buchnera* per bacteriocyte decreases to levels statistically indistinguishable from those found at or before the third instar. Coincident with the 3rd to 4th instar increase in *Buchnera* population size, the mean size of *Buchnera* cells also significantly increases (Figures 6C,D). While *Buchnera* population size at adulthood decreases to levels statistically indistinguishable from those found at or before the third instar, *Buchnera* cell size does not return to pre-reproductive sizes.

### Bacteriocyte Cells Increase in Size and Nuclear Area Across Aphid Post-embryonic Development

We imaged individual bacteriocytes as a single slice through the cell where the nucleus was visually evaluated to be the widest. For each bacteriocyte cell we measured the area of the cell and the area of the nucleus (Figures 6A,B). Bacteriocytes grow significantly between the third and fourth instar, and throughout adulthood variance in bacteriocyte cell size increases (Figure 6B). We found that as adult aphids age bacteriocytes increased in average area (*r*^2^ = 0.345, p = 0.004, slope = 359.4 ± 273.0) and became more variable in size (Kruskal-Wallis H = 1105.5, *p* = 2.35 × 10^–239^). We observed similar patterns across adulthood in the area (*r*^2^ = 0.419, p = 0.000419, slope = 359.4 ± 30.1) and variability in size (Kruskal-Wallis H = 37.424, *p* = 3.74 × 10^–8^) of the bacteriocyte nucleus. Bacteriocyte nuclei also increase in size across aphid post-embryonic development (Spearman’s ρ = 0.631, *p* = 2.84 × 10^–15^).

### Bacteriocyte Intracellular Environment Is Dynamic

**FIGURE 7:**
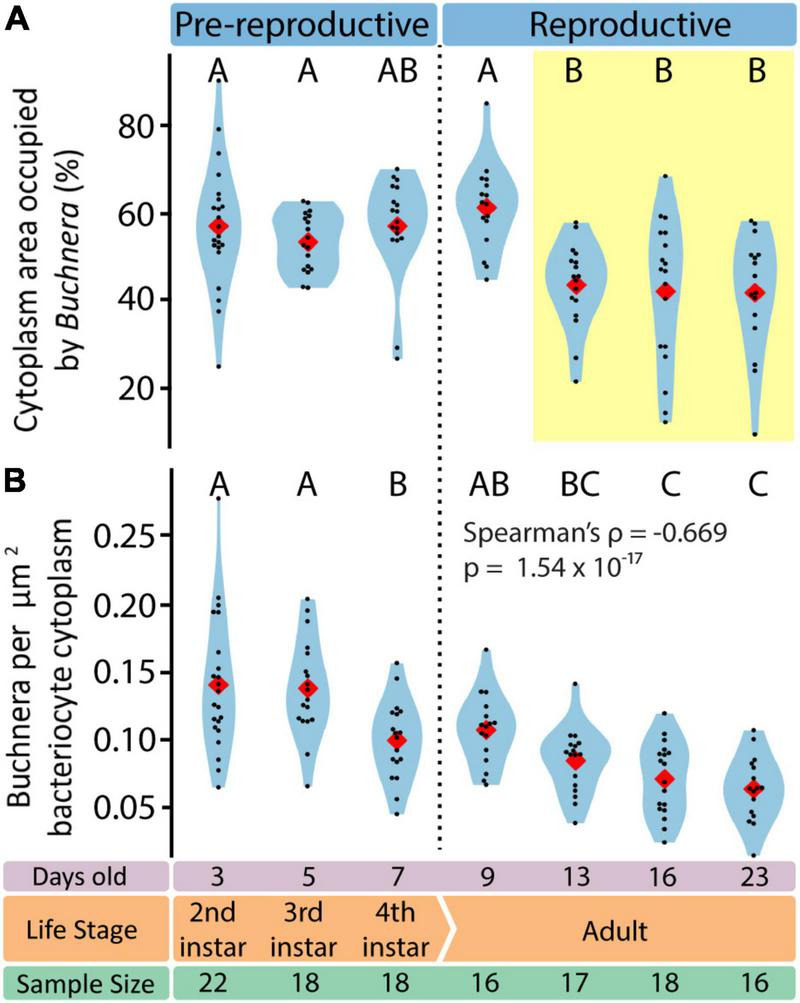
Endosymbiotic conditions vary dynamically throughout aphid life stage. **(A)** Percentage of bacteriocyte cytoplasm area occupied by *Buchnera.* Letters indicate Tukey *post hoc* grouping. **(B)** Density of *Buchnera* within aphid bacteriocytes in *Buchnera* per cytoplasm μm^2^. Letters indicate Dunn *post hoc* grouping with Benjamini/Hochberg correction. In all violin plots, red diamonds indicate means, black dots indicate individual data points.

To assess endosymbiotic conditions within bacteriocytes we recorded, for each bacteriocyte, *Buchnera* population density (*Buchnera* a μm^–2^), and the fraction of bacteriocyte cytoplasm occupied by *Buchnera* (% area). We found that *Buchnera* density decreased with aphid age (Spearman’s ρ = –0.669, *p* = 1.54 × 10^–17^) (Figure 7B) and that the area of cytoplasm occupied by *Buchnera* significantly decreased between day 9 and day 13 (Figure 7A) (ANOVA *F* = 7.79, *p* = 4.55 × 10^–7^). Notably, the significant decline in cytoplasmic space occupied by *Buchnera* was coincident with the time point at which empty vacuoles began to be found in bacteriocytes (Figure 4C).

## Discussion

### SymbiQuant Allows Novel Phenotyping of Endosymbiont Populations

SymbiQuant facilitates high-throughput study of endosymbiont populations in the context of their endosymbiotic tissue, allowing users to quantify changes in endosymbiont phenotype (size), and population dynamics across time or experimental treatment. Coupled with measurement of bacteriocyte cell and nuclei size using FIJI, SymbiQuant facilitates quantification of endosymbiont density metrics (Figure 7). In aphids, SymbiQuant adds novel bacteriocyte cell-level metrics to our current understanding of the aphid/*Buchnera* symbiosis based on insect-level methods like flow cytometry and genome-level methods like qPCR (Vogel and Moran, 2013; Simonet et al., 2016). By including a GUI to validate algorithm predictions we achieve expert-level high throughput bacteriocyte and *Buchnera* phenotyping, while saving a great deal of time compared to purely manual labeling.

Use of SymbiQuant in concert with current methods will enable new insights into the biology of symbiotic systems. It has previously been shown that genome copy number and population size are uncoupled in the aphid/*Buchnera* symbiosis (Komaki and Ishikawa, 2000), a pattern that holds true in other symbiotic systems (Mergaert et al., 2006; Woyke et al., 2010). SymbiQuant used alongside traditional qPCR approaches will allow for exploration of the relationship between *Buchnera* genome copy number and changes in biotic and abiotic conditions. For example it will be possible to ask how *Buchnera* genome copy number changes in response to nutritional input or how it varies across development. In combination with flow cytometry approaches, SymbiQuant can add cell-level information to complement insect-level data, allowing researchers to address questions about endosymbiotic systems at multiple levels of biological organization.

### Achieving a Synthetic Understanding of *Buchnera* Population Dynamics Across Embryonic Development

**FIGURE 8:**
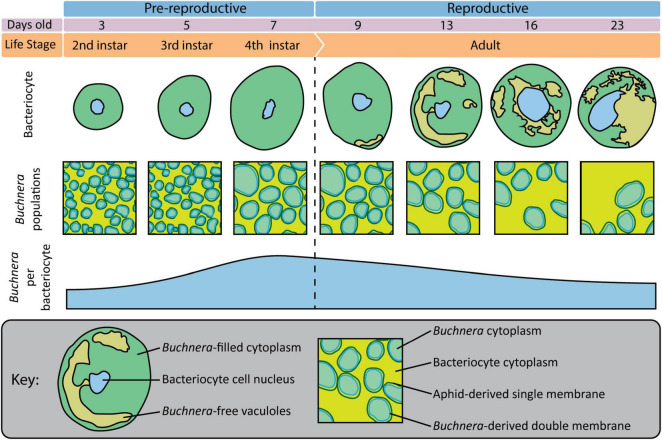
Overview of bacteriocytes and *Buchnera* populations throughout aphid postembryonic development.

Previously, using flow-cytometry Simonet et al. (2016) tracked changes in *Buchnera* populations over post-embryonic development in *A. pisum* line LL01. Here, using SymbiQuant and sampling at the same time points as Simonet et al. (2016), we tracked changes in *Buchnera* populations over post-embryonic development in *A. pisum* line LSR1. Both studies found that *Buchnera* population size increased significantly between the 3rd and 4th instar. Flow-cytometry, which quantifies *Buchnera* at the level of whole insect or whole tissue, revealed that *Buchnera* population size continued to increase into early adulthood, a result that we did not find at a bacteriocyte cellular-level (summarized in Figure 8). This disconnect between the whole insect level and bacteriocyte cell-level data suggests that *Buchnera* population size increases into young adulthood as a result of an increase in the number of bacteriocytes, and not an increase in the density of endosymbionts per host cell. Both studies also found that *Buchnera* density per bacteriocyte decreased over adulthood, with our observation of the presence of *Buchnera*-devoid vacuoles in bacteriocytes from day 13 onward being consistent with the timing of the bacteriocyte cell death process described in *A. pisum* LL01 by Simonet et al. (2018). In contrast, other patterns appear to differ between the two studies, for example, while Simonet et al. (2016) found that bacteriocytes significantly decreased in size in adults between day 16 and day 23, we did not observe a decrease in bacteriocyte cell size (Figure 6B). We reason that this difference between the two studies either reflects natural variation in the host and symbiont genotypes of aphid lines LL01 and LSR1, or could be attributed to other variables like differences in nutrition (Pers and Hansen, 2019). In addition to the work by Pers and Hansen demonstrating that *Buchnera* genome copy varies based on host stress, it has previously been shown that different *A. pisum* lines can have dramatically different *Buchnera* titers at any given developmental stage, and different patterns of *Buchnera* population growth through aphid life (Vogel and Moran, 2013; Chong and Moran, 2018).

Up to this point, quantification of *Buchnera* populations has largely been achieved using qPCR (e.g., Dunbar et al., 2007; Vogel and Moran, 2011; Chong and Moran, 2016; Enders and Miller, 2016; Zhang et al., 2016; Qian et al., 2018; Yao, 2019; Heyworth et al., 2020). Few studies have used absolute quantification (e.g., Plague et al., 2003), instead most have used relative quantification of a single copy *Buchnera* gene to a single copy aphid gene. Application of relative quantification assumes that the denominator is constant. Recent work by Nozaki and Shigenobu (2021) shows that the ploidy of bacteriocytes varies from 4 C to 512 C over *A. pisum* postembryonic development, and further is not consistent among bacteriocyte cells at any one time point in development. So, not only is *Buchnera* ploidy variable (Komaki and Ishikawa, 2000), so too is the ploidy of aphid bacteriocyte cells. This new discovery that both *Buchnera* and aphid cells show variable ploidy means that it is not possible to make meaningful comparisons between the work we present here and previous studies of the population dynamics of *Buchnera* as described by qPCR. Going forward it will be necessary for studies that make use of qPCR to use an absolute quantification approach to estimate *Buchnera* and aphid genome copy numbers. Further, we suggest that such estimates be made from dissected bacteriocytes and not whole insects. Application of absolute quantification approaches applied to symbiotic tissues will facilitate meaningful comparison of estimates of symbiont population size by genome copy counts vs. those based on whole cell analysis (i.e., flow cytometry and SymbiQuant).

### SymbiQuant Is Readily Adapted for Application in Other Endosymbiosis

**FIGURE 9:**
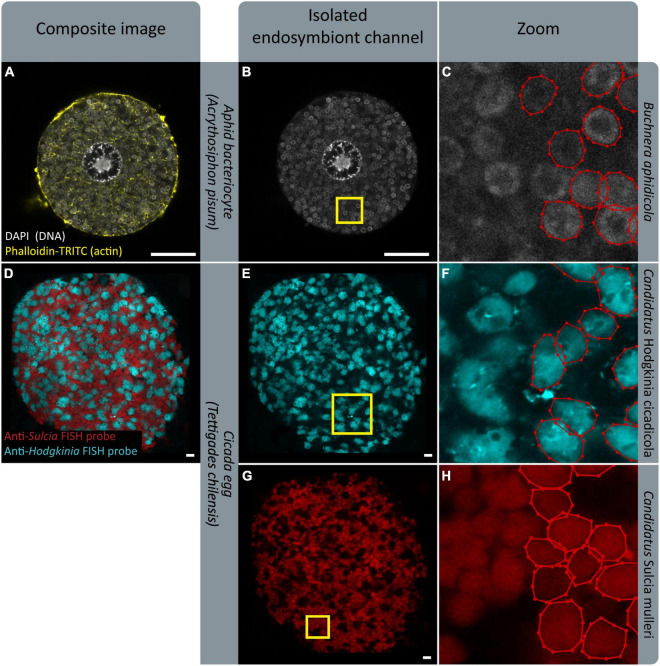
FISH gives endosymbiont-specific probes that work in complex symbiotic systems. Existing FISH data could serve as training datasets to implement PIPE in other systems. **(A,D)** Show confocal microscopy of endosymbiotic tissue; an aphid bacteriocyte, and a cicada egg, respectively. **(B,E,G)** Show isolated endosymbiont channels. **(B)** Shows signal from DAPI, staining endosymbiont genome DNA. **(E,G)** Show signal from endosymbiont-specific FISH probes. **(C,F,H)** Show zoomed sections indicated by yellow boxes in **(B,E,G)**, respectively. Endosymbionts in half of each panel have been labeled with labelme (Wada, 2016). (**D–H)** Show images provided by M. Campbell and J. McCutcheon, prepared in Campbell et al. (2018). **(D**,**E**,**G)** Show a microscope image overlaid on a black background for aesthetic purposes. Scale bars are 10 μm.

We designed SymbiQuant to return descriptive and quantitative data about endosymbiotic cells. Our group’s focal endosymbiosis is that of aphids and *Buchnera*, but in building SymbiQuant we worked to make it readily adaptable for application in other endosymbiotic systems. SymbiQuant can be adapted to annotate endosymbionts in any system in which it is possible to capture high resolution images of symbiotic tissue. Notably, by coupling image collection with taxon-specific labeling, SymbiQuant can even be applied in systems that include multiple endosymbionts (Figure 9). Previous work in the cicada *Tettigades chilensis* which is host to two endosymbionts (*Candidatus* Sulcia mulleri and *Candidatus* Hodgkinia cicadicola) used small-subunit rRNA probes to target each endosymbiont (Campbell et al., 2018). Using these probes Campbell et al. (2018) captured images of cicada oocytes packed with both *Sulcia* and *Hodgkinia*. In Figure 9 we show that by separating the image channels for each probe it would be possible to train SymbiQuant to “see” and count each endosymbiont. We include in the project GitHub^3^ a detailed description of how to train SymbiQuant for application in other endosymbiosis.

When Adapting SymbiQuant for application in other endosymbiotic systems, or when researchers use SymbiQuant to analyze images collected from aphid bacteriocytes it is important to develop a considered approach to the collection of microscopy data. Here by imaging each bacteriocyte one time at the point where the nucleus was widest we chose to invest resources in sampling many bacteriocytes, and describing the “average” bacteriocyte. In other studies, it may be more useful take a z-stack through each bacteriocyte being sure to start and end at consistent landmarks e.g., the “top” and “bottom” of the nucleus. Such an approach would provide a more comprehensive picture of each individual bacteriocyte but will be more costly. Additionally, if two different endosymbionts need to be analyzed from the same image as is shown in Figures 9D,E,G, we recommend collecting the signal from each endosymbiont in separate scans, using probes that do not overlap in their signal. Lastly, SymbiQuant was not designed to replace current approaches rather, coupled with existing approaches for quantifying endosymbiont populations it can provide additional insights into dynamics of endosymbiotic relationships.

### Region-Based Convolutional Neural Networks Have Broad Application in Biology

SymbiQuant adds to the use of region-based convolutional neural networks (RCNNs) for high-throughput processing of biological images. Recently, RCNNs have been implemented to record the number and morphology of stomata on leaves (Jayakody et al., 2021), to identify deep sea fauna (Liu and Wang, 2021), and even to track mouse movement (Geuther et al., 2019). We anticipate an increase in the use of RCNNs for capture of biological patterns and processes.

## Data Availability Statement

The datasets presented in this study can be found in online repositories. The names of the repository/repositories and accession number(s) can be found below: https://figshare.com/articles/online_resource/model_final_randomscale_0_5_1_40000_pth/19583713 and https://github.com/WilsonLabMiami/SymbiQuant.

## Author Contributions

EJ and AW conceived the project. EJ performed the lab work. EJ and XP wrote and executed the computational work with guidance from OS and AW. EJ and XP performed the data analysis with input from AW. EJ, XP, and AW drafted the manuscript. All authors read and contributed to revising the final version of the manuscript.

## Conflict of Interest

The authors declare that the research was conducted in the absence of any commercial or financial relationships that could be construed as a potential conflict of interest.

## Publisher’s Note

All claims expressed in this article are solely those of the authors and do not necessarily represent those of their affiliated organizations, or those of the publisher, the editors and the reviewers. Any product that may be evaluated in this article, or claim that may be made by its manufacturer, is not guaranteed or endorsed by the publisher.

## Footnotes

1
https://github.com/facebookresearch/detectron2

2
https://pytorch.org/

3
https://github.com/WilsonLabMiami/SymbiQuant
